# The pituitary tumour‐transforming gene 1/delta‐like homologue 1 pathway plays a key role in liver fibrogenesis

**DOI:** 10.1111/liv.15165

**Published:** 2022-01-30

**Authors:** Meritxell Perramón, Silvia Carvajal, Vedrana Reichenbach, Guillermo Fernández‐Varo, Loreto Boix, Laura Macias‐Muñoz, Pedro Melgar‐Lesmes, Jordi Bruix, Shlomo Melmed, Santiago Lamas, Wladimiro Jiménez

**Affiliations:** ^1^ Biochemistry and Molecular Genetics Service Hospital Clínic Universitari Barcelona Spain; ^2^ Institut d’Investigacions Biomèdiques August Pi i Sunyer (IDIBAPS) Centro de Investigación Biomédica en Red de Enfermedades Hepáticas y Digestivas (CIBEReHD) Barcelona Spain; ^3^ Department of Medicine University of Barcelona Barcelona Spain; ^4^ Barcelona‐Clínic Liver Cancer Group Hospital Clínic Universitari Barcelona Spain; ^5^ Department of Biomedicine University of Barcelona Barcelona Spain; ^6^ Institute for Medical Engineering and Science Massachusetts Institute of Technology Cambridge MA USA; ^7^ Department of Medicine, Cedars‐Sinai Research Institute University of California School of Medicine Los Angeles CA USA; ^8^ Centro de Biología Molecular Severo Ochoa (CSIC‐UAM) Madrid Spain

**Keywords:** extracellular matrix, fibrosis, gene therapy, liver, siRNA

## Abstract

**Background and Aims:**

*PTTG1* is almost undetectable in adult livers but is highly expressed in hepatocarcinoma. While little is known about its involvement in liver fibrosis, *PTTG1* expression is associated with *DLK1*. We assessed the role of the *PTTG1/DLK1* pathway in fibrosis progression and the potential therapeutic effect of *PTTG1* silencing in fibrosis.

**Methods:**

*Pttg1* and *Dlk1* were studied in liver and isolated cell populations of control and fibrotic rats and in human liver biopsies. The fibrotic molecular signature was analysed in *Pttg1* ^*−/−*^ and *Pttg1* ^*+/+*^ fibrotic mice. Finally, *Pttg1* silencing was evaluated in rats as a novel antifibrotic therapy.

**Results:**

*Pttg1* and *Dlk1* mRNA selectively increased in fibrotic rats paralleling fibrosis progression. Serum DLK1 concentrations correlated with hepatic collagen content and systemic and portal haemodynamics. Human cirrhotic livers showed greater *PTTG1* and *DLK1* transcript abundance than non‐cirrhotic, and reduced collagen was observed in *Pttg1* *Pttg1* ^*−/−*^ mice. The liver fibrotic molecular signature revealed lower expression of genes related to extracellular matrix remodelling including *Mmp*8 and *9* and *Timp4* and greater *eotaxin* and *Mmp13* than fibrotic *Pttg1* ^*+/+*^ mice. Finally, interfering *Pttg1* resulted in reduced liver fibrotic area, lower *α‐Sma* and decreased portal pressure than fibrotic animals. Furthermore, *Pttg1* silencing decreased the transcription of *Dlk1*, *collagens I* and *III*, *Pdgfrβ*, *Tgfrβ*, *Timp1*, *Timp2* and *Mmp2*.

**Conclusions:**

*Pttg1*/*Dlk1* are selectively overexpressed in the cirrhotic liver and participate in ECM turnover regulation. *Pttg1* disruption decreases *Dlk1* transcription and attenuates collagen deposition. *PTTG1/DLK1* signalling is a novel pathway for targeting the progression of liver fibrosis.

**ABBREVIATIONS**
Acta2, α‐SMA: alpha 2‐smooth muscle actin
Agt: angiotensinogen
AII: angiotensin II
Akt1: AKT serine/threonine kinase 1
ANOVA: one‐way analysis of variance
Bcl2: B‐cell lymphoma 2
bwt: body weight
C^−^ siRNA: negative control siRNA
Cav1: Caveolin 1
Ccl11: C–C motif chemokine ligand 11
Ccl3: C–C motif chemokine ligand 3
CCl_4_
: carbon tetrachloride
Ccr2: C–C motif chemokine receptor 2
Col1a2: collagen type I Alpha 2 Chain
Col3a1: collagen type III Alpha 1 Chain
Ctgf: cellular communication network factor 2
Cxcr4: C–X–C motif chemokine receptor 4
ddPCR: droplet digital PCR
DLK1: delta‐like homologue 1
EC: endothelial cell
ECM: extracellular matrix
Edn1: endothelin 1
Egf: epidermal growth factor
Fasl: tumour necrosis factor receptor superfamily, member 6 ligand
Grem1: gremlin 1
HCC: hepatocellular carcinoma
HDAC: histone deacetylases
HEP: hepatocytes
HIF1: hypoxia‐inducible factor 1
HSC: hepatic stellate cells
Ifng: interferon Gamma
Il: interleukin
Il13ra2: interleukin 13 receptor subunit alpha 2
Ilk: integrin linked kinase
Inhbe: inhibin subunit beta E
Itga2: integrin subunit alpha 2
Itgb: integrin subunit beta
KO: knock out
Lox: lysyl oxidase
MAP: mean arterial pressure
MMPs: matrix metalloproteinases
Myc: MYC proto‐oncogene, BHLH, transcription factor
Pdgf: platelet‐derived growth factor
Plat: plasminogen activator, tissue type
Plau: plasminogen activator, urokinase
PP: portal pressure
*Pttg1* siRNA: PTTG1 pre‐designed siRNA
PTTG1: pituitary tumour‐transforming gene 1
RT‐PCR: real‐time PCR
siRNA: small interference RNA
Smad3: SMAD family member 3
Smad6: SMAD family member 6
Smad7: SMAD family member 7
Stat6: signal transducer and activator of transcription 6
TACE: tumour necrosis factor‐alpha converting enzyme
Tgfbr: transforming growth factor‐beta receptor
TGFβ: transforming growth factor‐beta
Thbs: thrombospondin
TIMPs: tissue inhibitor of matrix metalloproteinases
TNFα: tumour necrosis factor‐alpha
WT: wild type

## Lay Summary

*PTTG1* and *DLK1* transcription are increased in rats and patients with hepatic cirrhosis. *PTTG1* is involved in fibrotic extracellular matrix remodeling and its silencing decreases portal hypertension and alliviates fibrosis progresion.

## 1 INTRODUCTION

Cirrhosis is a major determinant of morbidity and mortality and predisposes to hepatic failure and liver cancer. Halting the progression of fibrosis to cirrhosis is considered as a foremost goal in patients with liver disease. Anti‐inflammatory agents, arresting hepatic stellate cells (HSC) activation substances, renin‐angiotensin system inhibitors, cannabinoid receptor antagonists, hepatoprotective peptides, transforming growth factor‐β (TGFβ) or platelet‐derived growth factor (PDGF) antagonists and chemokine receptor antagonists are among the numerous candidates assessed to limit or reverse liver fibrogenesis.^1^, ^2^, ^3^, ^4^ However, most of these compounds have shown limited efficacy and/or adverse side effects, and consequently, an antifibrogenic pharmacological treatment for liver fibrosis is currently lacking.

The pituitary tumour‐transforming gene (*PTTG1)* is the index mammalian securin.^5^ *PTTG1* is overexpressed in a variety of cell lines including hepatocellular carcinoma (HCC).^6^ It encodes a multifunctional protein involved in the regulation of faithful chromatid segregation during mitosis, DNA repair, apoptosis, metabolism and gene transcription.^7^ Interestingly, *PTTG1* modulates extracellular matrix (ECM) turnover regulating several matrix metalloproteinases (MMPs).^8^, ^9^ Despite overexpression of *PTTG1* in liver biopsies from patients with HCC, very little data are available on its expression in preneoplastic conditions such as advanced liver fibrosis and cirrhosis. This is particularly striking as several factors induce *PTTG1* expression, including estrogens, fibroblast growth factor, insulin, insulin growth factor‐1 and hepatocyte growth factor^7^, ^10^ all increased under conditions of chronic liver injury.^11^, ^12^, ^13^, ^14^ Moreover, micro‐environmental hypoxia occurring in damaged hepatic tissue could also regulate *PTTG1* expression through the hypoxia‐inducible factor 1.^15^ *PTTG1* also acts to regulate growth factors, angiogenesis and exhibits transforming activity *in vitro* and in vivo.^16^, ^17^ Furthermore, hepatic *PTTG1* expression is upregulated after partial hepatectomy and has been proposed as a new marker of proliferation in liver regeneration.^18^ These findings support the exploration of whether *PTTG1* could contribute to the activation of fibroproliferative processes in liver disease. In addition, delta‐like homologue 1 (*DLK1*) was identified as one of the most abundantly expressed *PTTG1* targets.^19^ The *DLK1* gene encodes a single‐pass transmembrane protein that belongs to a family of epidermal growth factor (EGF) repeat‐containing proteins.^20^ DLK1 is a non‐canonical ligand of Notch receptors that mediate a metabolic shift from lipid storage to peripheral lipid oxidation in adipocytes, participate in differentiation processes and behave as a growth factor.^21^ It consists of six EGF‐like tandem repeats, a juxtamembrane region with a tumour necrosis factor‐alpha converting enzyme (TACE)‐mediated cleavage site, a transmembrane domain and a short intracellular tail.^22^ DLK1 can act as both transmembrane and soluble protein. DLK1 membrane‐proximal cleavage by TACE results in the release of the EGF‐like extracellular region, a large soluble product of 50 kDa. This form has a similar function inhibiting adipocyte differentiation to that of the full‐length membrane‐associated protein, but since it is soluble it can act in an autocrine and paracrine manner. Moreover, both *PTTG1* and *DLK1* genes show concomitant expression in human fetal liver, placenta and different carcinomas, including pituitary adenoma, breast adenocarcinoma and neuroblastoma.^19^ Given this background, we aimed to explore the hypothesis that *PTTG1/DLK1* signalling should play a central role in the activation of the fibrogenic process in liver disease.

## 2 MATERIALS AND METHODS

### 2.1 Induction of hepatic cirrhosis in rats

This study was performed in control (n = 32) and male Wistar rats with different degrees of fibrosis (n = 77) (Charles‐River, Saint Aubin Les Elseuf, France). Fibrosis was induced by repetitive carbon tetrachloride (CCl_4_) inhalation.^23^ The rats were fed ad libitum with standard chow and water containing phenobarbital (0.3 g/L), as drinking fluid. Animals were exposed to a CCl_4_ atmosphere twice a week, starting with 0.5 minutes for three sessions. Afterwards, the duration was increased to 1, 2, 3, 4 and 5 minutes until the end of the investigation. To induce variable degrees of hepatic fibrosis CCl_4_‐treated rats were studied at the 8th, 13th, 16th and 19th week after starting the fibrosis induction protocol. Control rats were studied following similar periods of phenobarbital administration. When scheduled, animals were anaesthetised and a haemodynamic study was performed. Afterwards, a blood sample was obtained and animals were sacrificed by isoflurane overdose (Forane, Abbott Laboratories S.A., Madrid, Spain). Organ samples were snap‐frozen or fixed in 10% buffered formalin.

### 2.2 Induction of fibrosis in mice

This study was performed in fibrotic and control male *Pttg1* wild‐type (*Pttg1* ^*+/+*^) and knock out (*Pttg1* ^*−/−*^ *)* mice. *Pttg1* ^*−/−*^ mice with C57BL/6 genetic background were provided by Dr Shlomo Melmed and the origin of these mice has been described previously.^24^ Fibrosis was induced in *Pttg1* ^*+/+*^ (n = 4) and *Pttg1* ^*−/−*^ (n = 7) by i.p. injection of CCl_4_ (1 ml CCl_4_/kg of body weight [bwt], previously diluted 1:8 vol/vol in corn oil) three times a week for 4 weeks. All animals were kept under constant temperature and humidity in 12 hours controlled dark/light cycle, and they were fed ad libitum on a standard pellet diet.

### 2.3 In vivo *Pttg1* interference

A group of fibrotic rats randomly received i.v. *Pttg1* small‐interfering RNA (siRNA, assay ID s133880, 0.25 mg/kg/dose bwt, n = 6) or scrambled siRNA (Ambion in vivo negative control No. 1, n = 6) as the negative control (C^−^ siRNA) every 10 days from the 9th to the 13th week after starting the fibrosis induction protocol. In vivo transfection was performed using Invivofectamine 3.0 kit (Invitrogen, Life Technologies Corporation, Carlsbad, CA, USA) following the manufacturer’s instructions. Six control rats were also included. Rats were studied in the 14th week.

### 2.4 Statistical analysis

Quantitative data were analysed using GraphPad Prism 5 (GraphPad Software Inc., San Diego, CA) and statistical analysis of the results was performed by unpaired Student’s *t* test, one‐way analysis of variance (ANOVA) with Newman‐Keuls post hoc test or Kruskal‐Wallis test with Dunn post hoc test when appropriate. Correlations between the variables studied were analysed with Pearson two‐tailed test. Results are expressed as mean ± SE and considered significant with *P* < 0.05.

Additional materials and methods are provided in the Supplementary material section.

## 3 RESULTS

### 3.1 Fibrosis quantification and staging

**FIGURE 1:**
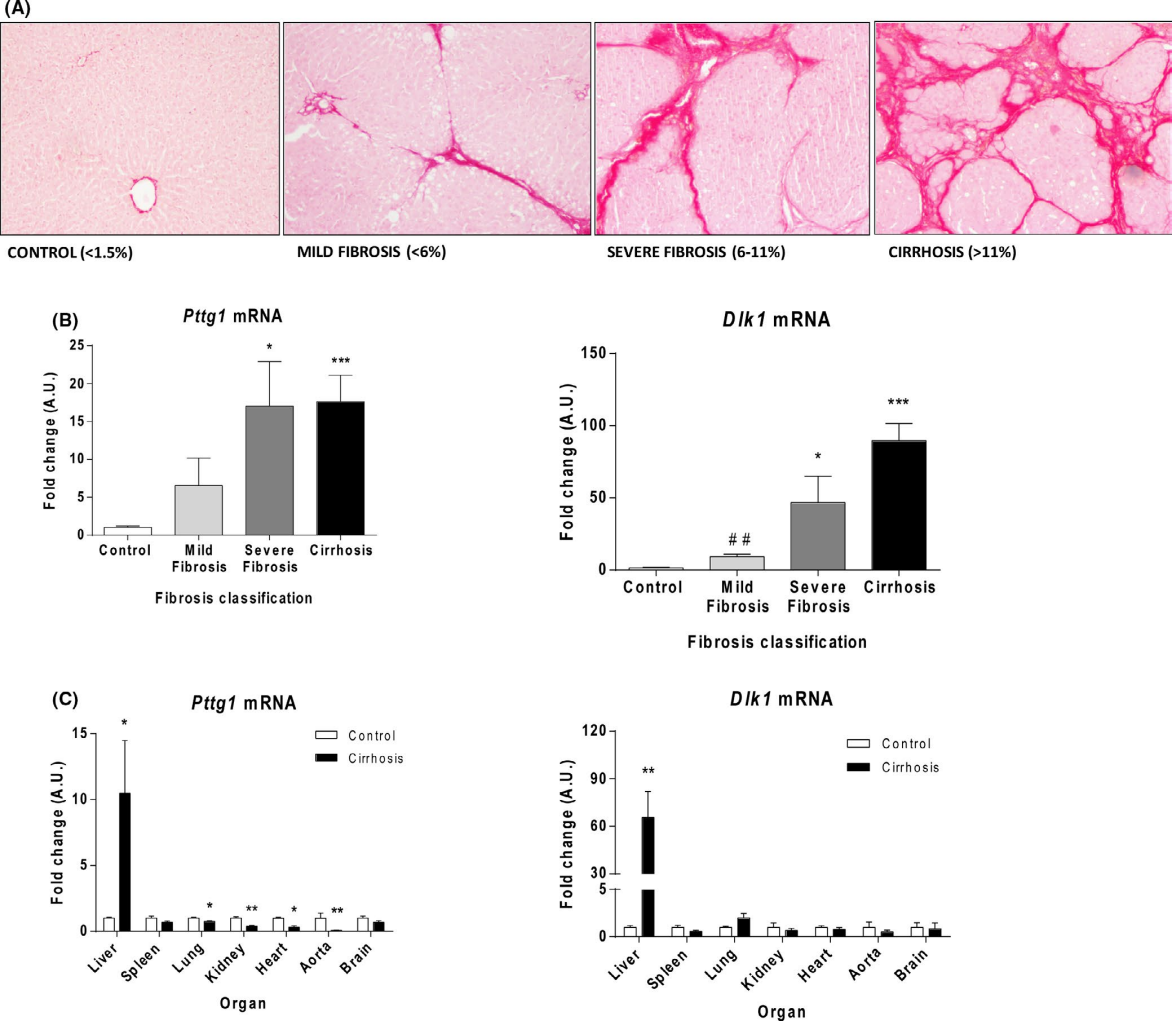
Expression of *Pttg1* and *Dlk1* in experimental liver fibrosis. A, Staging of CCl_4_‐treated rats based on liver‐collagen content. Sirius red staining of representative liver sections (×100). B, Hepatic *Pttg1* and *Dlk1* mRNA of control (n = 13) and CCl_4_‐treated rats with mild/moderate fibrosis (n = 6), severe fibrosis (n = 8) and cirrhosis (n = 11). C, *Pttg1* and *Dlk1* mRNA in organs from control (n = 5) and cirrhotic rats (n = 5). Results are expressed as mean ± SE. *P <  0.05, **P <  0.01, ***P < 0.001 vs control; ^##^ *P <* 0.01 vs cirrhosis. One‐way ANOVA with Newman‐Keuls post hoc test or Kruskal‐Wallis test with Dunn post hoc test

The liver of rats treated with CCl_4_ showed macroscopic finely granulated surfaces. According to the time of CCl_4_ exposure, we observed progressive ECM accumulation, evolving from a light deposition, mainly in the portal area, to numerous and thicker septa in those animals submitted to longer CCl_4_ exposure periods. Most animals exposed to the toxin for the longest periods of time developed cirrhosis. Consequently, rats were staged according to the percentage of fibrotic area with respect to the total area of the liver biopsy: mild and moderate fibrosis was defined when the percentage of fibrotic area <6% (n = 6), severe fibrosis 6%–11%, (n = 8) and cirrhosis >11% (n = 11). Control rats (n = 13) displayed no appreciable alterations in liver histology. Figure 1A shows representative Sirius red staining from a control liver, a liver with mild/moderate fibrosis, a liver with severe fibrosis and a cirrhotic liver. Fibrotic/cirrhotic rats had important alterations in liver function tests, which were more pronounced in cirrhosis (Table S1).

### 3.2 Hepatic *Pttg1* and *Dlk1* mRNAs parallel the intensity of liver fibrosis and selectively occurs in this organ

Progression of liver fibrosis was associated with a concomitant increase in *Pttg1* mRNA expression (Figure 1B). *Pttg1* expression significantly increased in rats with severe fibrosis and reached maximum levels in rats with cirrhosis. Interestingly, *Pttg1* transcript was selectively detected in the liver of cirrhotic animals, but not in the spleen, lungs, kidneys, heart, aorta or brain (Figure 1C). *Pttg1* mRNA abundance was also assessed by droplet digital PCR (ddPCR). Results were in line with those obtained in real‐time PCR (RT‐PCR) experiments. The liver of cirrhotic rats showed a much higher abundance of *Pttg1* transcripts (411 ± 67 copies /μl) than that found in control livers (20 ± 2 copies /μl, *P* ˂  0.001). In contrast, with the exception of heart (7 ± 1 vs 28 ± 2 copies/μl, *P* ˂  0.05) no differences were found between spleen (614 ± 94 vs 947 ± 172 copies/μl), kidney (27 ± 4 vs 38 ± 5 copies /μL), lung (74 ± 8 vs 87 ± 5 copies/μl) aorta (4 ± 1 vs 27 ± 12 copies /μL) and brain (17 ± 3 vs 21 ± 1 copies /μl) of cirrhotic and control rats. The pattern expression of *Pttg1* was paralleled by a similar profile for *Dlk1* mRNA (Figure 1B). *Dlk1* mRNA abundance progressively increased, the lowest levels observed in rats with mild/moderate fibrosis, the highest in cirrhotic rats. Indeed, *Dlk1* activation was selectively detected in the cirrhotic liver but not in other assessed organs (Figure 1C).

### 3.3 *Pttg1* and *Dlk1* are mainly expressed in hepatic parenchymal tissue

**FIGURE 2:**
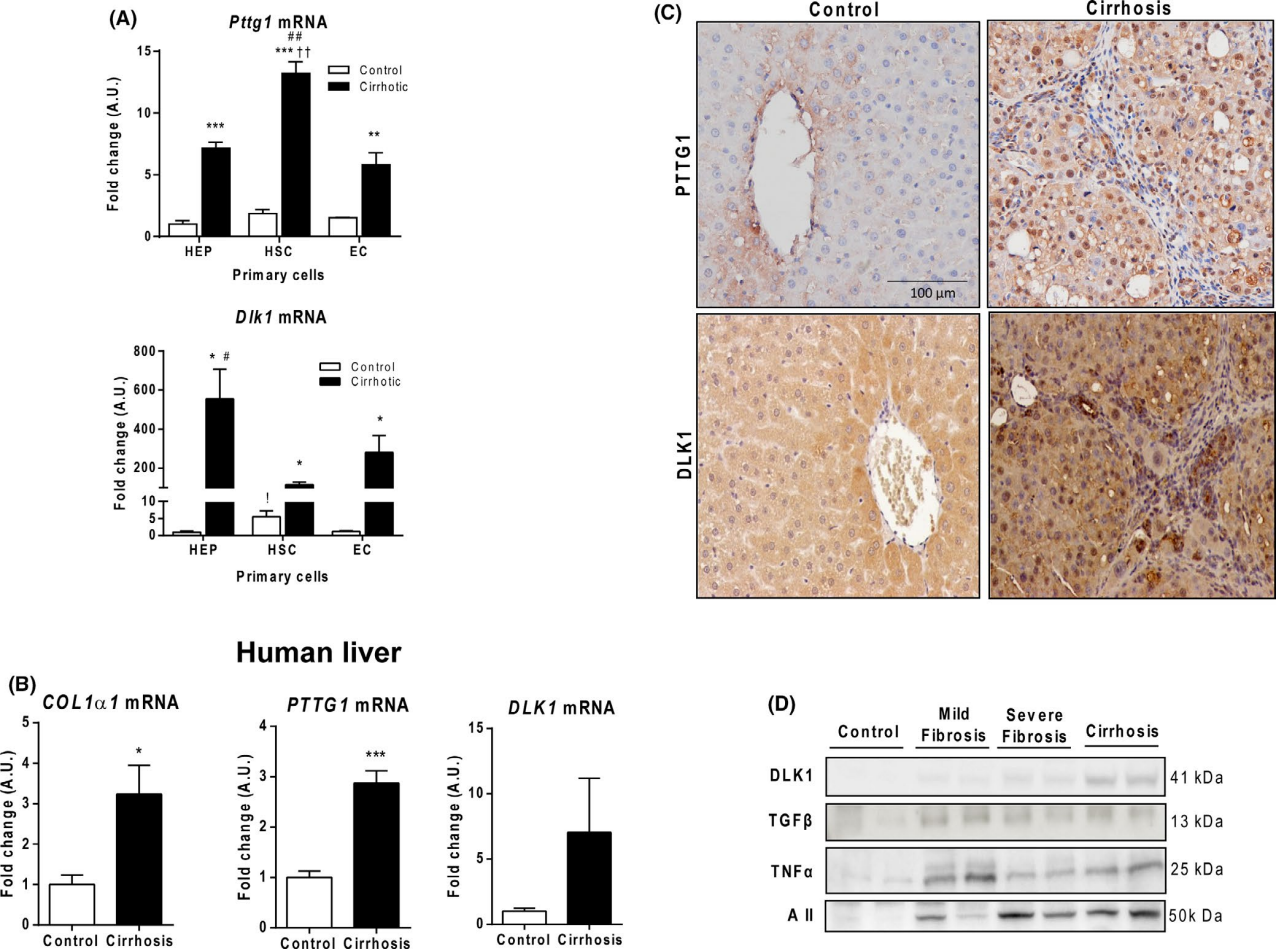
*Pttg1* and *Dlk1* expression. A, *Pttg1* and *Dlk1* mRNA in hepatocytes (HEP), stellate cells (HSC) and endothelial cells (EC) from control (n = 2) and cirrhotic rat livers (n = 2). Results are expressed as mean ± S.E. *P <  0.05, **P <  0.01 vs control, ††*P <*  0.01 vs cirrhotic HEP; ^#^ *P <*  0.05, ^##^ *P* 0.01 vs cirrhotic EC. !*P* <  0.05 vs control HEP. B, *COL1α1*, *PTTG1* and *DLK1* mRNA in liver from cirrhotic (n = 12) and non‐cirrhotic patients (n = 7). *P <  0.05, ***P <  0.001 vs control. C, Immunnolocalization of PTTG1 and DLK1 in rat control and cirrhotic liver (200×). D, Western blots for rat hepatic DLK1, TGFβ, TNFα and angiotensin II (AII)

To identify the cellular source of altered expression of both *Pttg1* and *Dlk1* in hepatic tissue, we isolated primary cells from the liver of cirrhotic and control rats. Both, *Pttg1* and *Dlk1* exhibited low or almost negligible mRNA expression in different control cell types (Figure 2A). By contrast, marked *Pttg1* mRNA abundance was observed in three types of liver cells isolated in cirrhotic rats, the highest abundance being found in HSC (Figure 2A). This was paralleled by striking activation of *Dlk1* mRNA but was largely observed in hepatocytes (HEP) (Figure 2A). In an attempt to further delineate the relative contribution of HEP and HSC to the acute increase in *Pttg1* and *Dlk1* in cirrhotic liver, we next measured the absolute concentration of these transcripts in the isolated cells. In line with the RT‐PCR results, the absolute *Pttg1* mRNA values were similar in both types of cells (HEP: 125 ± 7 copies /μl, HSC: 131 ± 3 copies /μl), whereas *Dlk1* mRNA values were lower in HSC (80 ± 11 copies /μl) than in HEP (154 ± 9 copies /μl). Overactivation of the PTTG1/DLK1 axis in human cirrhosis was further confirmed. Paralleling the increased abundance of collagen I alpha 1 (*COL1α1)* messenger, higher expression of both *PTTG1* and *DLK1* mRNA was observed in samples derived from cirrhotic patients in comparison to non‐cirrhotic biopsies. Next, we performed histological immunolocalization of PTTG1 and DLK1 in the liver of cirrhotic and control rats. Both proteins were almost undetectable in control samples. However, in cirrhotic livers, they were clearly identified either in the parenchymal area or close to the portal tracts and fibrous septa (Figure 2C). Additionally, DLK1 expression clearly differs from that of other well established profibrogenic substances, since DLK1 hepatic protein content only exhibited a clear relationship with fibrosis intensity (Figure 2D).

### 3.4 Serum DLK1 values rise in parallel with liver fibrosis and correlate with hemodynamics

**FIGURE 3:**
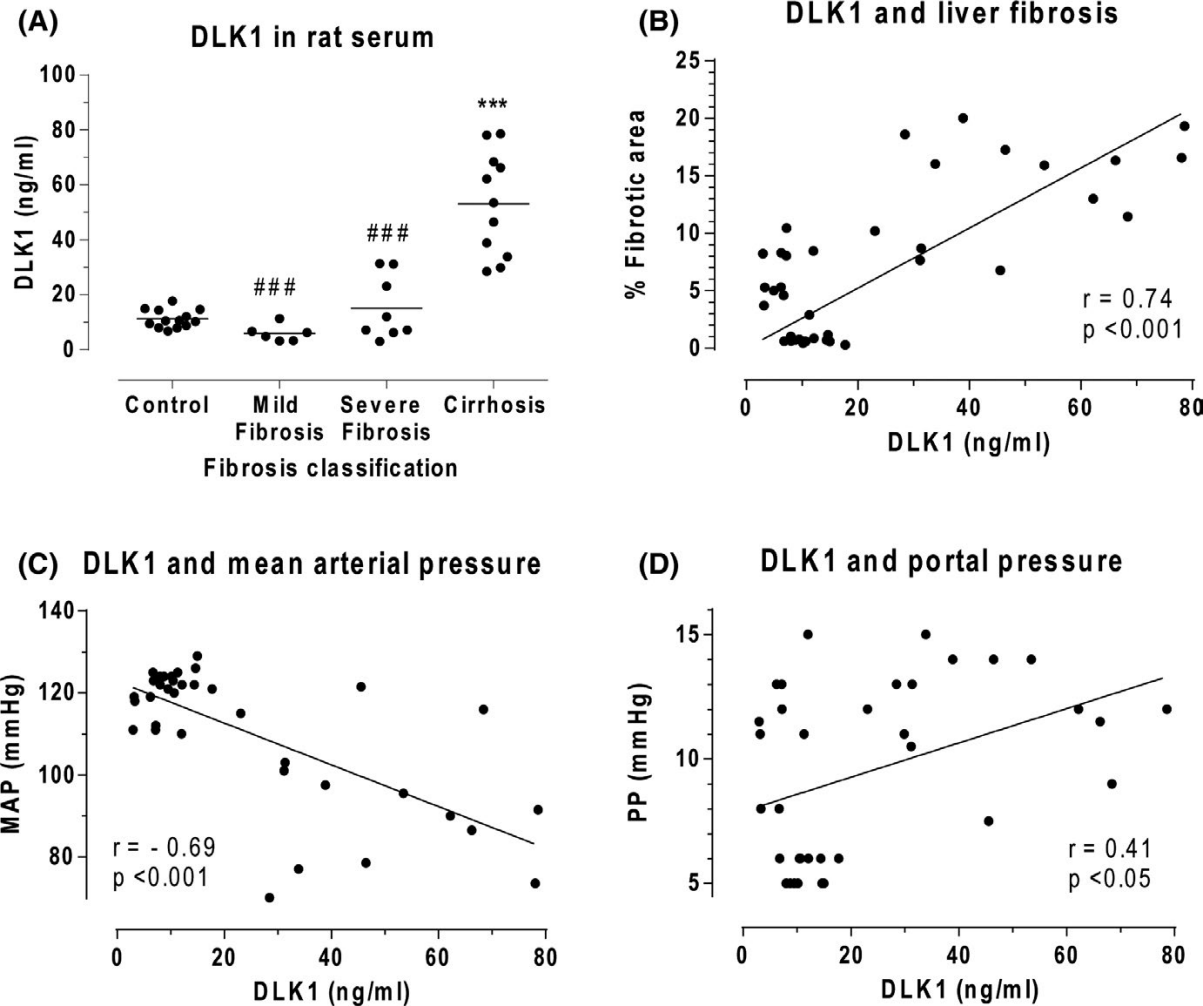
DLK1 serum levels in rats with experimental fibrosis. A, Serum concentrations of DLK1 in control (n = 13), mild/moderate fibrosis (n = 6), severe fibrosis (n = 8) and cirrhotic (n = 11) rats. Each point represents single DLK1 value in serum. Horizontal lines indicate the mean value for each group. ***P <  0.001 vs control; ^###^ *P*  < 0.001 vs cirrhotic. One‐way ANOVA with Newman‐Keuls post hoc test. Correlation of DLK1 serum levels with (B) histological quantification of liver fibrosis (*r* = 0.74; *P* < 0.001); C mean arterial pressure (*r* = −0.69; *P* <  0.001; and D, portal pressure (*r* = 0.43; *P* <  0.01) in control (n = 13) and CCl_4_‐treated (n = 25) rats. Pearson two‐tailed test

Advanced progression of liver fibrosis was associated with a parallel increase in circulating levels of DLK1, showing a significant increase in rats with cirrhosis (53.13 ± 5.66 ng/ml, *P* <  0.001) (Figure 3A). A close direct relationship between DLK1 and hepatic collagen content was found in CCl_4_‐treated rats (*r* = 0.74; *P* <  0.001) (Figure 3B). Animals with cirrhosis showed frank hypotension (mean arterial pressure [MAP]: 87 ± 13 mm Hg, *P* < 0.001) as compared with control rats (MAP: 123 ± 2 mm Hg). MAP inversely correlated with serum DLK1 in CCl_4_‐treated rats (*r* = −0.69, *P* <  0.001) (Figure 3C). Furthermore, serum concentration of DLK1 also depicted a direct relationship with the degree of portal hypertension in fibrotic/cirrhotic animals (*r* = 0.41, *P* < 0.05) (Figure 3D).

### 3.5 Fibrosis is significantly attenuated in Pttg1^*−/−*^ mice

**FIGURE 4:**
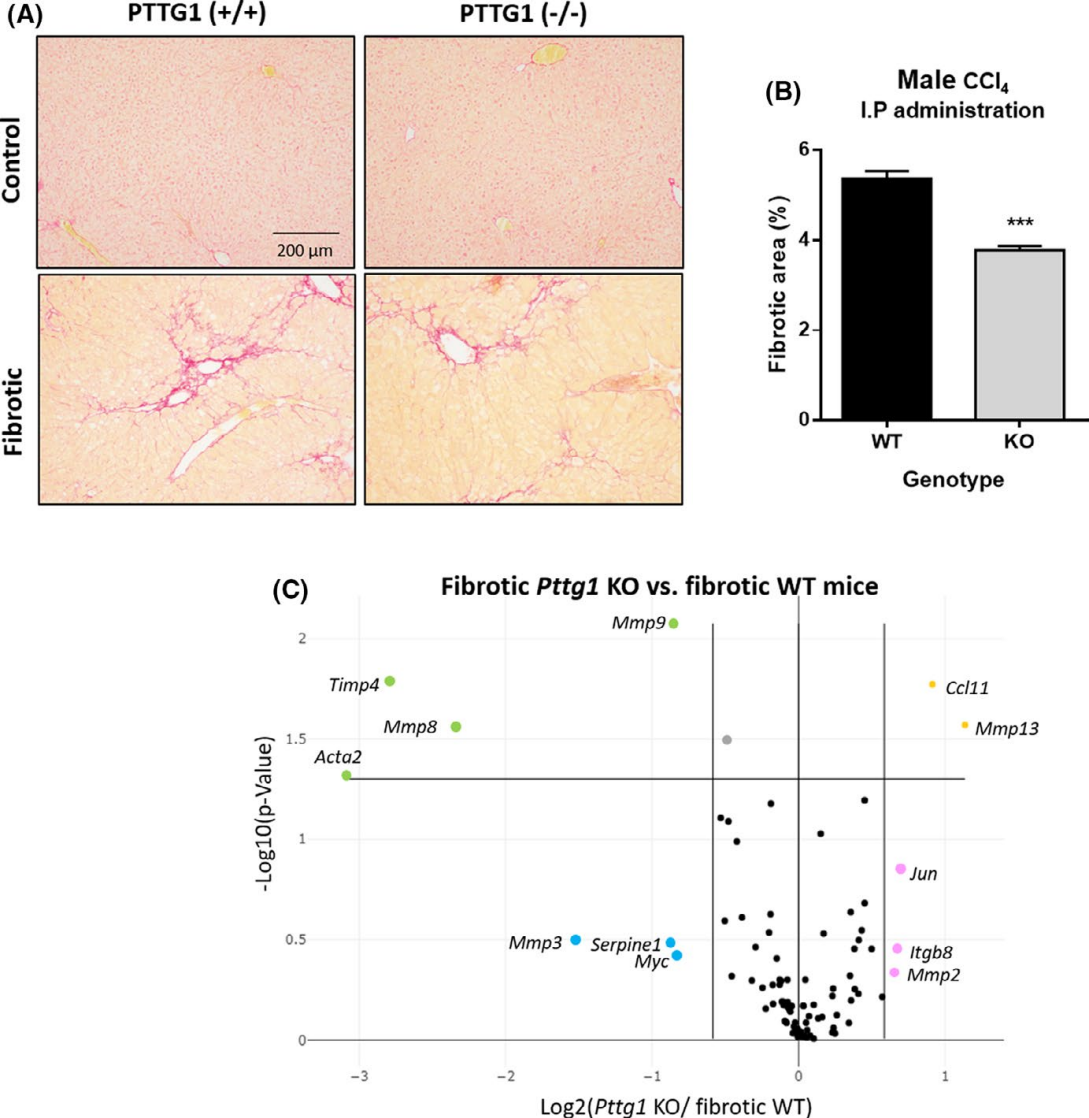
Hepatic fibrosis in *Pttg1^*−/−*^* mice. A, Sirius red staining from healthy and fibrotic *Pttg1* ^*+/+*^ and *Pttg1* ^*−/−*^ mice (100×). B, Fiber content in *Pttg1* ^*+/+*^ (n = 4) and *Pttg1* ^‐/‐^ (n = 7) mice. ***P ≤ 0.001 vs *Pttg1* ^*+/+*^, unpaired *t*‐test. C, Volcano plot of the differentially expressed genes in a pair‐wise comparison of *Pttg1* ^*+/+*^(n = 4) and *Pttg1* ^‐/‐^ (n = 4) mice. Significance was set to a *P* value based on a Student’s *t*‐test of 0.05 (−log10 [*P*‐value] ≥1.30), the biological cut‐off was set to a fold regulation of ±1.5 fold (−1 ≥log1.5 [FC of fibrotic Pttg1 KO/fibrotic WT] ≥1). According with these two criteria, the top 12 differentially expressed genes are labeled with their corresponding gene ID. Insignificant (black), statistically but not biologically significant down‐regulated (grey), biologically but not statistically downregulated (blue) and upregulated (pink), and both biologically and statistically significant downregulated (green) up regulated (yellow) genes in fibrotic *Pttg1* ^*+/+*^ mice

CCl_4_‐treated *Pttg1* ^*+/+*^ mice had mild/moderate fibrosis mainly characterized by perivenular and periportal deposition with incipient development of the portal and venular septa, ending blindly in the parenchyma; whereas *Pttg1* ^*−/−*^ mice displayed thinner septa and more preserved hepatic parenchyma (Figure 4A) than *Pttg1* ^*+/+*^ animals. These findings were confirmed by morphometric analysis in which *Pttg1* ^*−/−*^ mice showed a significantly reduced percentage of fibrosis area than sections of *Pttg1* ^*+/+*^ mice (Figure 4B). This attenuation in liver fibrosis was also associated with an almost 50% reduction in *Dlk1* mRNA expression. In fact, whereas fibrotic *Pttg1* ^*+/+*^ mice showed a 55.7 ± 5.0‐fold change increase in *Dlk1* mRNA overcontrol *Pttg1* ^*−/−*^ mice, these figures were of a 30.9 ± 7.5‐fold change increase in *Pttg1* ^*−/−*^ fibrotic mice.

### 3.6 Expression pattern of fibrogenesis‐related genes in *Pttg1* ^*−/−*^ mice

Further insight on the effects of *Pttg1* ^*−/−*^ in the liver of CCl_4_‐treated mice was obtained by determining the mRNA expression of 86 genes involved in the fibrogenic process. Table 1 shows all the genes showing a 1.5‐fold or greater change in expression between the liver of *Pttg1* ^*+/+*^ control or CCl_4_‐treated mice. Regardless of the moderate hepatic collagen deposition observed in *Pttg1* ^*+/+*^ mice treated with CCl_4_, these animals showed a clear fibrotic molecular signature. Actually, 24 genes were significantly upregulated, including *Acta2* and *Grem1* which encode proteins involved in HSC activation and epithelial to mesenchymal transition,^25^ genes related to ECM and adhesion molecules (*Col1a2*, *Col3a1*, *Mmp1a*, *Mmp9* and *Plau*) and several genes related to inflammation (*Il1α*, *Il1β*, *Ilk, Il10, Tnf, Ccl3* and *Cxcr4*), growth (*Agt*) and signal transduction (*Inhbe, Smad3, Stat6, Smad6, Tgfb1, Tgfbr1, Tgfbr2, Thbs1* and *Thbs2*) compared to control *Pttg1* ^*+/+*^ mice.

**TABLE 1:** Hepatic mRNA expression of genes involved in pathogenic mechanisms of liver fibrosis showing 1.5‐fold or greater regulation between control *Pttg1* ^*+/+*^(n = 4) and fibrotic *Pttg1* ^*+/+*^(n = 4) mice

Gene symbol	Fold regulation	Gene symbol	Fold regulation
Fibrosis			
*Acta2*	1.92**	*Bcl2*	2.35
*Grem1*	−2.35*	*Fasl*	1.78
Extracellular matrix and cell adhesion molecules			
*Col1a2*	4.72***	*Mmp9*	4.44**
*Col3a1*	3.79***	*Mmp13*	5.59
*Lox*	2.95	*Mmp14*	2.42
*Itga2*	2.38	*Plat*	7.52
*Itgb3*	1.57	*Plau*	4.50*
*Itgb5*	1.57	*Serpine1*	9.35
*Mmp1a*	4.54**	*Timp1*	2.84
*Mmp2*	8.51	*Timp2*	2.06
*Mmp3*	1.55	*Timp3*	1.86
*Mmp8*	4.55	*Timp4*	1.96
Inflammatory cytokines and chemokines			
*Ccl3*	7.58*	*Il1a*	2.98**
*Ccl11*	−1.75	*Il1b*	2.69*
*Ccr2*	4.35	*Il4*	−1.56
*Cxcr4*	3.44*	*Il5*	−1.61
*Ifng*	2.38	*Ilk*	1.53***
*Il10*	4.43*	*Tnf*	4.64*
*Il13ra2*	1.82		
Growth factors			
*Agt*	1.60**	*Egf*	1.73
*Ctgf*	−1.65	*Pdgfa*	1.72
*Edn1*	1.52	*Pdgfb*	2.43
Signal transduction			
*Cav1*	1.51	*Tgfb1*	2.04**
*Inhbe*	2.85	*Tgfb2*	2.67
*Myc*	2.69	*Tgfb3*	2.69
*Smad3*	1.83*	*Tgfbr1*	1.79*
*Smad6*	2.17*	*Tgfbr2*	2.37*
*Smad7*	1.91	*Thbs1*	3.01*
*Stat6*	1.69*	*Thbs2*	2.98*
Epithelial‐to‐mesenchymal transition			
*Akt1*	1.63		

Abbreviations: *mRNA* determined by *Acta2,* Alpha 2 smooth muscle actin*; Agt,* angiotensinogen; *Akt1*, AKT serine/threonine kinase 1; *Bcl2*, B‐cell lymphoma 2; *Cav1*, caveolin 1; *Ccl3,* C–C Motif chemokine ligand 3; *Ccl11,* C–C motif chemokine ligand 11; *Ccr2*, C–C motif chemokine receptor 2; *Col1a2*, collagen type I Alpha 2 Chain; *Col3a1*, collagen type III Alpha 1 Chain; *Ctgf*, cellular communication network factor 2; *Cxcr4*, C–X–C motif chemokine receptor 4; *Edn1*, Endothelin 1; *Egf*, epidermal growth factor; *Fasl*, tumour necrosis factor receptor superfamily, member 6 Ligand; *Grem1*, Gremlin 1; *Ifng*, interferon gamma; *Il1a*, Interleukin 1 Alpha; *Il1b*, Interleukin 1 Beta; *Il4*, Interleukin 4; *Il5*, Interleukin 5; *Il10*, Interleukin 10; *Il13ra2*, Interleukin 13 Receptor Subunit Alpha 2; *Ilk*, Integrin Linked Kinase; *Inhbe*, Inhibin Subunit Beta E; *Itga2*, Integrin Subunit Alpha 2; *Itgb3*, Integrin Subunit Beta 3; *Itgb5*, Integrin Subunit Beta 5; *Lox*, Lysyl Oxidase; *Mmp1a*, Matrix Metallopeptidase 1a; *Mmp2*, matrix metallopeptidase 2; *Mmp3*, matrix metallopeptidase 3; *Mmp8*, matrix metallopeptidase 8; *Mmp9*, matrix metallopeptidase 9; Mmp13, matrix metallopeptidase 13; *Mmp14*, matrix metallopeptidase 14; *Myc*, MYC proto‐oncogene, BHLH transcription factor; *Pdgfa*, platelet‐derived growth factor subunit A; *Pdgfb*, platelet‐derived growth factor subunit B; *Plat*, plasminogen activator, tissue type; *Plau*, plasminogen activator, Urokinase; *Serpine1*, serpin family E member 1; *Smad3*, SMAD family member 3; *Smad6*, SMAD family member 6; *Smad7*, SMAD family member 7; *Stat6*, signal transducer and activator of transcription 6; *Tgfb1*, transforming growth factor‐beta 1; *Tgfb2*, transforming growth factor‐beta 2; *Tgfb3*, transforming growth factor‐beta 3; *Tgfbr1*, transforming growth factor‐beta receptor 1; *Tgfbr2*, transforming growth factor‐beta receptor 2; *Thbs1*, thrombospondin 1; *Thbs2*, thrombospondin 2; *Timp1*, tissue inhibitor of metallopeptidase 1; *Timp2*, tissue inhibitor of metallopeptidase 2; *Timp3*, tissue inhibitor of metallopeptidase 3; *Timp4*, tissue inhibitor of metallopeptidase 4; *Tnf*, tumour necrosis factor.

* *P <*  0.05

** *P <*  0.01

*** *P* <  0.001 vs control WT mice. Unpaired Student’s *t* test.

A 1.5‐fold or greater change in expression with *P* <  0.05 was considered statistically significant in comparing fibrotic *Pttg1* ^*+/+*^ vs *Pttg1* ^*−/−*^ mice. In addition to reducing the mRNA expression of *Acta2* (gene encoding α‐smooth muscle actin protein), the lack of *Pttg1* inhibited the expression of several genes involved in ECM turnover including *Mmp8*, *Mmp9* and *Timp4* (Figure 4C). We also observed a significant increase in *Ccl11* and *Mmp13*. The former encodes eotaxin a chemokine that has been described to be upregulated in senescent HSC^26^ whereas the latter encodes for a metalloprotease involved in the degradation of a fibrotic liver matrix.^27^

### 3.7 Assessment of *Pttg1* siRNA in cultured rat hepatocytes and fibrotic rats

**FIGURE 5:**
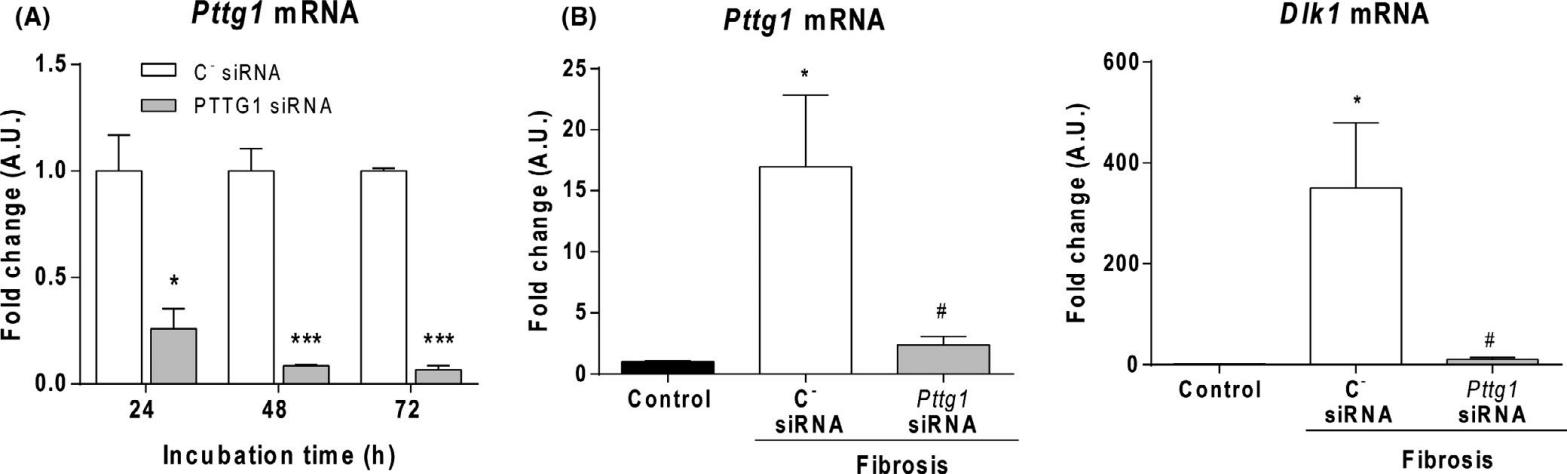
Effect of *Pttg1* siRNA on *Pttg1* and *Dlk1* expression. A, mRNA expression of *Pttg1* in CC‐1 cells transfected with C^‐^siRNA or *Pttg1* siRNA for 24, 48 and 72 h. Results are expressed as mean ± SE. *P <  0.05, ***P <  0.001 vs C^‐^siRNA. One‐way ANOVA with Newman‐Keuls post hoc test. B, mRNA xpression of *Pttg1* and *Dlk1* in liver tissue of control (n = 6) and fibrotic rats treated with C^‐^siRNA (n = 6) or *Pttg1* siRNA (n = 6). *P <  0.05 vs C^‐^siRNA; ^#^ *P* < 0.05 vs *Pttg1* siRNA. Kruskal‐Wallis test with Dunn post hoc test

To investigate the efficacy and duration of gene silencing in cultured rat hepatocytes, we transfected CC‐1 cells. Following treatment, *Pttg1* mRNA was significantly lower than that in the siRNA C^−^ group at 24, 48 and 72 hours (Figure 5A). These results indicate that *Pttg1 siRNA* effectively suppresses *Pttg1* expression in rat‐cultured hepatocytes. *Pttg1* siRNA was also effective at silencing the enhanced expression of *Pttg1* mRNA in fibrotic rats (Figure 5B). In fact, whereas fibrotic rats treated with scrambled siRNA displayed approximately 15 times higher levels of *Pttg1* mRNA than control animals, an abundance of this transcript in the liver of fibrotic rats receiving *Pttg1 siRNA* was not different from that found in healthy animals. In addition to silencing hepatic *Pttg1* mRNA, administration of *Pttg1* siRNA also inhibited hepatic *Dlk1* mRNA expression in fibrotic rats (Figure 5B).

### 3.8 Effect of *Pttg1* siRNA on liver histology, portal pressure and profibrogenic genes in fibrotic rats

**FIGURE 6:**
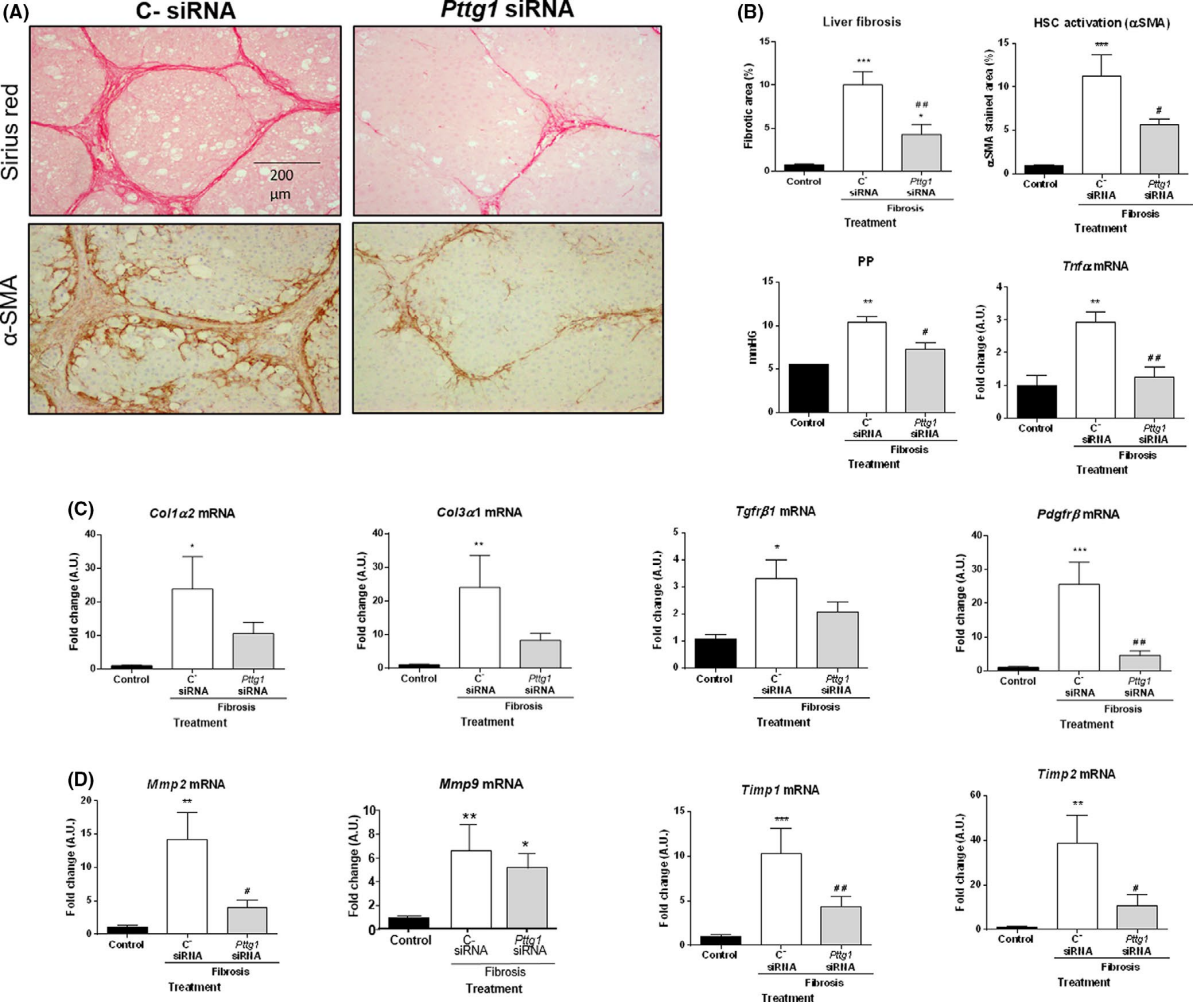
Effect of *Pttg1* blockade on fibrosis progression. Control (n = 6) and fibrotic rats receiving C^‐^siRNA (n =6) or *Pttg1* siRNA (n = 6). A, Sirius red and α‐SMA stainings (100×). B, Quantitative measurement of relative fibrosis and α‐SMA positive area, portal pressure and *Tnfα* mRNA. C, Hepatic messenger expression of *Col1α2*, *Col3α1*, *Tgfβr1* and *Pdgfrβ*. D, Hepatic *Mmp2*, *Mmp9*, *Timp1* and *Timp2* mRNA expression. Results are given as mean ± SE. *P <  0.05, **P <  0.01, ***P <  0.001 vs control; ^#^ *P* <  0.05, ^##^ *P* <  0.01 vs C^‐^siRNA treated rats. One‐way ANOVA with Newman‐Keuls post hoc test or the Kruskal‐Wallis test with Dunn post hoc test

Fibrotic rats treated with C^−^ siRNA showed initial stages of the characteristic pattern of perivenular and periportal deposition of connecting tissue with development of portal‐to‐portal septa and evidence of architectural distortion resulting in micronodular fibrosis (Figure 6A). However, biopsies obtained from fibrotic rats treated with *Pttg1* siRNA displayed less remarkable architectural alterations, with thinner septa, and more preserved hepatic parenchyma. This was confirmed by morphometric analysis of Sirius red‐stained sections (Figure 6B). This abrogation of fibrosis was consistently observed in all animals exposed to *Pttg1* silencing. Similar results were found when staining alpha 2 smooth muscle actin (α‐SMA). We detected α‐SMA as linear staining in the portal tracts and fibrous septa of both groups of fibrotic rats (Figure 6A). Staining was more diffuse in rats receiving *Pttg1* siRNA than in C^−^ siRNA. In line, *Pttg1* siRNA also showed significantly reduced portal hypertension than fibrotic animals receiving C^−^ siRNA (Figure 6B). Furthermore, *Pttg1*‐silenced animals significantly decreased hepatic mRNA expression of *Tnfα* compared to fibrotic rats.

As anticipated, *Col1α2* and *Col3α1* mRNA was significantly increased in fibrotic rats treated with C^−^ siRNA. Consistently, we also observed activation of key genes involved in profibrogenic mechanisms, such as *Tgfßr1* and *Pdgfrβ* (Figure 6C), and an altered balance of MMPs and TIMPs, specifically, increased transcription of *Mmp2*, *Mmp9, Timp1* and *Timp2* (Figure 6D). In line with previous results, administration of *Pttg1* siRNA resulted in a significantly lower abundance of *Col1α2*, *Col3α1, Mmp2*, *Timp1* and *Timp2* transcripts (Figure 6C,D). Furthermore, *Tgfßr1* and *Pdgfrß* expression appeared also markedly attenuated, indicating that *Pttg1* silencing effectively abrogates profibrogenic activity in CCl_4_‐induced fibrosis.

### 3.9 Effect of *Pttg1* siRNA on serum markers of liver function

*Pttg1* siRNA treatment was associated with a tendency towards normalization of most systemic indicators of liver function (Table 2). Actually, aspartate aminotransferase, lactate dehydrogenase, gamma‐glutamyl transferase, total bilirubin and triglycerides were found to be near normal. Overall, these results support the protective effects on hepatic function resulting from *Pttg1* mRNA silencing in rats with experimental fibrosis.

**TABLE 2:** Serum markers of liver function in control rats and fibrotic rats treated with C^−^ or *Pttg1* siRNAs

	Control (n = 6)	Fibrosis	
		C^−^ siRNA (n = 6)	*Pttg1* siRNA (n = 6)
Alanine transaminase (U/L)	43 ± 6	1069 ± 356*	1226 ± 385*
Aspartate transaminase (U/L)	56 ± 11	2342 ± 561**	924 ± 235
Lactate dehydrogenase (U/L)	356 ± 27	1197 ± 224*	349 ± 49
Gamma‐glutamyl transferase (U/L)	0.03 ± 0.03	5.38 ± 1.68**	2.40 ± 1.10
Total bilirubin (mg/dL)	0.00 ± 0.00	1.08 ± 0.32***	0.22 ± 0.12
Total proteins (g/L)	49.7 ± 2.2	41.0 ± 3.0*	44.5 ± 1.8
Albumin (g/L)	27.6 ± 1.8	23.9 ± 1.9	27.0 ± 2.1
Total cholesterol (mg/dL)	55.4 ± 9.9	68.29 ± 8.39	50.80 ± 6.43
Triglycerides (mg/dL)	4.34 ± 1.03	57.5 ± 12.7**	33.0 ± 7.7
Glucose (mg/dL)	207 ± 36	73 ± 23*	91 ± 21

One‐way ANOVA with the Newman–Keuls post hoc test or the Kruskal‐Wallis test with the Dunn post hoc test when appropriate. Results are given as mean ± SE.

* *P* <  0.05

** *P* <  0.01

*** *P* < 0.001 vs control.

## 4 DISCUSSION

This investigation aimed to explore whether *PTTG1/DLK1* signalling contributes to the activation of the fibroproliferative process in liver disease. In agreement with previous studies, *Pttg1* mRNA was almost undetectable in healthy animals. In contrast, *Pttg1* mRNA levels were markedly overexpressed in the liver of rats with hepatic fibrosis, reaching maximal abundance in cirrhotic rats. Moreover, only hepatic tissue of cirrhotic rats showed a significantly increased abundance of *Pttg1* mRNA with respect to control animals. Paralleling *Pttg1* results, *Dlk1* mRNA expression markedly increased in fibrotic rats, with a close correlation with collagen deposition. *Dlk1* transcript was also selectively overexpressed only in the liver. In addition, liver DLK1 protein levels mirrored expression patterns of the cognate transcript. The lowest abundance was found in rats with mild fibrosis and the highest in cirrhotic rats. This clearly differs from the behaviour of other profibrogenic factors. Cell fractionation experiments showed increased *Pttg1* and *Dlk1* mRNA in HEP, HSCs and endothelial cells (ECs) in liver tissue of cirrhotic rats compared to controls. Furthermore, PTTG1 and DLK1 protein staining in cirrhotic rats resulted in an intense, although topologically undefined, positive signal in the hepatic parenchyma, being more pronounced close to the portal tracts. Finally, on exploring the expression of *PTTG1*/*DLK1* in the human liver we also observed that, whereas control livers showed negligible *PTTG1* or *DLK1* mRNA expression, samples from cirrhotic patients markedly overexpressed *PTTG1* mRNA. To our knowledge, this is the first investigation demonstrating *PTTG1* mRNA induction in human cirrhosis. In parallel, *DLK1* was also increased in human samples in line with previous investigations showing that *DLK1* is frequently upregulated in human HCC but rarely detected in adjacent non‐cancerous liver tissue.^28^ Overall, our findings indicate that the *PTTG1/DLK1* pathway is relevant in the pathogenesis of liver fibrosis. This contention was further supported by results obtained in *Pttg1* KO mice. Previous investigations performed in thioacetamide‐induced fibrosis showed significantly weaker macromorphological signs of bridging fibrosis in *Pttg1* ^*−/−*^ in comparison to *Pttg1* ^*+/+*^ mice.^29^ This finding was further confirmed in the current study in CCl_4_‐treated mice, indicating that the lack of *Pttg1* attenuates the development of murine liver fibrosis. Subsequent gene expression analysis pointed toward disruption of ECM turnover as a major driver of this phenomenon. In fact, stringent analysis of alterations in gene expression induced by *Pttg1* ^*−/−*^ in fibrotic animals, considering only those genes showing both statistically and biologically significant downregulation, revealed a group of genes all involved in fibrogenesis including *Mmp9, Mmp8, Timp4* and *Acta2*. Of interest, this occurred in the setting of diminished *Myc* mRNA expression, which encodes a nuclear phosphoprotein that regulates the transcription of numerous genes involved in the cell cycle, cell growth, differentiation, apoptosis, transformation, genomic stability and angiogenesis.^30^ Moreover, *Pttg1* is a powerful activator of *Myc,* ^31^ which suggest that this gene could play a central role in *PTTG1* induced fibrosis signalling pathway.

Our findings suggest that RNA‐based therapy targeting *Pttg1*, such as siRNA, may prevent the development of liver fibrosis. siRNA‐*Pttg1‐*treated rats displayed significantly weaker macromorphological signs of liver fibrosis, a decrease in portal hypertension and a lesser amount of activated HSC. These results suggest that a reduction in the proportion of activated HSC is involved in the inhibition of liver fibrogenesis. Amelioration in portal pressure is most likely a consequence of the antifibrotic effect induced by *Pttg1* siRNA administration. These results are also supported by a lower abundance of liver *Col1α2* and *Col3α1* mRNAs and some other markers of active fibrosis such as *Tgfβr1* and *Pdgfrβ*. It has been reported that interference of *PTTG1* in ovarian epithelial tumour cells resulted in diminished expression and release of TGFβ, whereas increased expression of *Pttg1* mRNA mirrored *Tgfβ* mRNA expression.^32^ In our study *Pttg1* mRNA interference tended towards lower levels of *Tgfβr1,* although without statistical significance. This is consistent with previous reports in fibrotic *Pttg1* null mice in which hepatic *Tgfβ* mRNA was significantly lower than in the liver of WT mice.^29^

*Pttg1* mRNA interference in fibrotic rats also downregulated hepatic expression of the PDGF receptor, *Pdgfrβ*. Specific blockade of the intrahepatic PDGFRβ pathway with adenoviral vectors in CCl_4_‐induced fibrosis rats^33^ or systemic PDGF antagonism in bile duct ligated rats led to significantly reduced hepatic fibrosis.^34^ Considering that PDGF is the most potent pro‐proliferative cytokine for HSCs^35^, ^36^ reduced fibrosis observed after *Pttg1* mRNA interference treatment likely is a consequence of blockade of HSC proliferation and inhibition of chemotaxis, which thereby decreases the number of cells able to synthesize ECM proteins.

Administration of *Pttg1* siRNA to fibrotic rats also affects the regulation of ECM remodelling. In experimental and human cirrhosis, fibrosis appears to be the result of not only excessive ECM synthesis but also reduced degradation.^37^, ^38^ Fibrotic rats receiving C^−^ siRNA presented a marked induction of *Col1α2* and *Col3α1* gene expression as well as a significant upregulation of *Mmp2* and *Mmp9* which can be the result of a compensatory mechanism designed to eliminate the excess of scar tissue. In our study, treatment with *Pttg1* siRNA was also associated with a significant increase in the MMPs/TIMPS ratio. This could be due to preferential *Timp1* and *Timp2* inhibition, thereby supporting the concept that PTTG1 activity is a regulator of the ECM degradation pattern in the injured liver mainly by controlling TIMPs activity.

Interestingly, we also observed a tendency towards normalization of most surrogate liver function serum markers. However, this was not the case with transaminases. This finding, previously documented in null *Pttg1* fibrotic mice,^29^ indicates that *Pttg1* mRNA interference has no effect on CCl_4_‐induced hepatotoxicity and further supports the concept that *Pttg1* deficiency directly interferes with fibrosis.

Several potential mechanisms mediate the effects of *Pttg1* knockdown on hepatic fibrosis. First, the *Pttg1* blockade prevents hepatic *Dlk1* overexpression. *Pttg1* acts as a post‐transcriptional regulator of *Dlk1,* ^19^ and *Dlk1* inhibition results in reduced HSC activation and associated fibrosis.^39^ The decrease of *Dlk1* expression mediated by *Pttg1* downregulation was associated with a significant reduction in activation of HSC, suggesting that PTTG1/DLK1 pathway plays a pivotal role in the hepatic fibroproliferative process. On the other hand, both *Pttg1* and *Dlk1* share common regulatory mechanisms in which histone deacetylases (HDAC) are involved. Actually, HDAC1 expression has been shown to be responsible for the proliferation of corticotroph cells via *Pttg1,* ^40^ whereas HDAC3 activity represses *Dlk1* expression in preadipocytes and NIH 3 T3 cells.^41^ Second, *Pttg1* is involved in the regulation of ECM turnover key proteins. Experimental evidence indicates that *Pttg1* is a major regulator of *Mmp2* by inducing its secretion and expression.^9^ MMP2 plays an important role in remodelling basement membranes as it degrades several components including collagen IV, laminin and fibronectin.^38^ The present study shows that *Mmp2* expression increases in liver fibrosis, however, *Pttg1* interference may have an antifibrogenic effect also by reducing *Mmp2* expression and, consequently, by blocking degradation of normal perisinusoidal matrix and promoting activation of quiescent HSC.^42^ In this study, we also observed that *Pttg1* blocking reduced TIMPs expression. *Timp1* and T*imp2* are mainly expressed in activated HSC, thus, *Timp1* and *Timp2* expression could be reduced as a result of diminished HSC activation in these animals. TIMPs also stimulate fibroblast proliferation.^43^ Thus, *Timp1* and *Timp2* downregulation could also contribute to decreased proliferation of activated HSC. A graphical model summarizing the proposed mechanism underlying PTTG1‐induced promotion of liver fibrosis is provided in the supplementary information section.

In conclusion, this investigation shows that serial administration of *Pttg1* siRNA exerts antifibrotic effects when administered during induction of hepatic damage. *Pttg1* gene silencing normalizes expression of *Dlk1*, arrests activation of HSC, diminishes expression of ECM‐related genes and finally decreases hepatic collagen deposition and reduces portal hypertension. Thus, the PTTG1/DLK1 axis may represent a valuable target for the prevention and treatment of liver fibrosis.

## CONFLICT OF INTEREST

The authors declare no competing interests. Dr Bruix consults for, advises, and is on the speakers’ bureau and received grants from Bayer‐Shering and BTG. He consults for and advises MSD. He consults for and is on the speakers’ bureau for Sirtex. He consults for and received grants from Arqule and Ipsen. He consults for Novartis, Bristol‐Myers Squibb, Eisai, Kowa, Terumo, Gilead, Bio Alliance, Roche, AbbVie, Merck, AstraZeneca, Incyte, Quirem, Adaptimmune and Lilly.

## ETHICAL APPROVAL

All animal procedures and human samples were approved by the Investigation and Ethics Committee of the Hospital Clinic and Animal Experimentation Committee of the University of Barcelona (Barcelona, Spain). Human samples have consented for research in accordance with ethical guidelines of the 1975 Declaration of Helsinki.

## Supporting information


Appendix S1

## Data Availability

The data that support the findings of this study are available from the corresponding author upon reasonable request.
