# Supplementary material for: The pituitary tumour‐transforming gene 1/delta‐like homologue 1 pathway plays a key role in liver fibrogenesis
Source: Liver Int. 2022 Jan 30;42(3):651–62. doi: 10.1111/liv.15165 (PMC9303549; doi:10.1111/liv.15165)
Supplement: Supplementary file 1 — Appendix S1 [file LIV-42-651-s001.docx]

**THE pituitary tumor-transforming gene 1 (PTTG1) / Delta like homolog 1 (DLK1) PATHWAY PLAYS A KEY ROLE IN LIVER FIBROGENESIS**

Meritxell Perramón, Silvia Carvajal, Vedrana Reichenbach, Guillermo Fernández-Varo, Loreto Boix, Laura Macias, Pedro Melgar-Lesmes, Jordi Bruix, Shlomo Melmed, Santiago Lamas, Wladimiro Jiménez

**Table of contents**

Supplementary Materials & methods...................................................................2

Supplementary graphical model .......................................................................13

Supplementary Table 1 .....................................................................................15

References .......................................................................................................16

**Supplementary Materials & methods**

***Mouse genotyping***. Mouse genomic DNA was isolated from ear biopsies using a specific kit (Extract-N-Amp™ Tissue PCR Kit; Sigma-Aldrich, Darmstadt, Germany). PCR was performed using the primer pairs to amplify the *Pttg1* gene (primer forward: *5’-*GTGCTACTTCCATTTGTCACGTCC-3’ and primer reverse: 5’-TTAGCTGTGAGCTCGTCGGTG-3’) and other primer pairs in order to verify the disruption of the *Pttg1* gene (primer forward: 5’-TAGGCTTTTCGGCAACTCTGTTGAC-3’ and primer reverse: 5’-TTCTGGGGACTGAATTCAGG-3’). The PCR conditions were as follows: 38 cycles at 94 °C for 30 s, 58 °C for 1 min, and 72°C for 1 min 40 s. PCR products were electrophoresed in 1.5 % agarose 1x TAE (40 mM Tris, 20 mM acetic acid and 1 mM ethylendiaminetetraacetic acid) gels. For visualization, gels were stained with 1×SYBR® Safe DNA Gel Stain (Life Technologies) and digital images were captured in ImageQuant™ LAS 4000 to distinguish the WT (220 bp) and *Pttg1* KO (700 bp) mice.

***Messenger expression of Dlk1 and fibrosis gene expression PCR array in the liver of Pttg1 KO mice*.** Total RNA of control WT (n=4), fibrotic WT (n=4), and fibrotic *Pttg1* KO (n=4) mice was extracted using a RNA extraction column kit (RNAeasy, Qiagen, Venlo, The Netherlands). RNA concentrations were determined by spectrophotometric analysis (ND-100 spectrophotometer; Thermo Fisher Scientific, Waltham, MA, USA). First strand cDNA was synthesized from 500 ng of total RNA using a RT^2^ First Strand Kit (Qiagen). Primers and probes for gene expression assays (Applied Biosystems) were selected as follows: *Dlk1* (Taqman assay reference from Applied Biosystems: Mm00494477_m1) and *Hprt* used as an endogenous standard (Mm03024075_m1). Expression assays were designed using the Taqman Gene Expression assay software (Applied Biosystems). RT-PCR was analyzed in duplicate and performed with a Lightcycler-480 II (Roche Diagnostics). Real-time PCR arrays were performed using the RT² Profiler™ PCR Array Mouse Fibrosis (Qiagen). All procedures were performed according to the manufacturer’s protocol. This PCR array combines the quantitative performance of SYBR Green-based Real-time PCR with the multiple gene profiling capabilities of microarrays to profile the expression of 86 key genes involved in fibrogenic processes. PCR array plates were processed in a Light Cycler 480 (Roche Diagnostics) using automated baseline and threshold cycle detection. Gene expression was normalized to internal controls to determine the fold change in gene expression between test and control samples. The relative quantity of the product was expressed as fold-induction of the target gene compared with the reference gene according to the formula 2^-ΔΔCT^. Data were interpreted using the SABiosciences web-based PCR array data analysis tool (http://pcrdataanalysis.sabiosciences.com/pcr/arrayanalysis.php).

***Hemodynamic measurements.*** Rats were anesthetized with Inactin® (100 mg/kg bwt, Sigma-Aldrich Chemie Gmbh, Steinherim, Germany) and prepared with a PE-50 polyvinyl catheter in the left femoral artery. A blood sample (1 ml) was obtained from each animal to analyze standard biochemistry tests. A midline abdominal incision (2 cm) was made and the portal vein was cannulated through an ileocolic vein with a PE-50 catheter to measure PP. After verifying free blood reflux, the catheter was fixed to the mesentery with cyanoacrylate glue. Hemodynamic parameters were recorded in a multichannel system (PowerLab®, ADInstruments, Sydney, Australia). Hemodynamic parameters were allowed to equilibrate for 30 min and values of MAP and PP were recorded over two time periods of 10 min. Each value represents the average of 2 measurements.

***Fibrosis quantification.*** Liver sections (4 µm) were stained in 0.1 % Sirius red F3B (Sigma-Aldrich, St. Louis, MO, USA) in saturated picric acid (Sigma-Aldrich). Sirius red selectively binds collagen proteins and was used to stain collagen fibrils in the liver of CCl_4_-treated rats (1). The relative fibrotic area, expressed as a percentage of total liver area, was assessed by analyzing 32 fields of Sirius red-stained liver sections per animal as previously described (2). Each field was acquired at 100X magnification with an E600 microscope (Nikon, Tokyo, Japan) and a RT-Slider Spot digital camera (Diagnostic Instruments, Sterling Heights, MI, USA). Results were analyzed using imaging software (ImageJ, NIH). To evaluate the relative fibrosis area, the collagen area measured was divided by the net field area and then multiplied by 100. Subtraction of the vascular luminal area from the total field area yielded the net fibrosis area.

***Liver cell fractionation.*** Primary hepatocytes, liver endothelial cells (EC), and HSCs were freshly isolated from the livers of control (n= 2) and cirrhotic (n= 2) adult male Wistar rats. Animals were euthanized by isoflurane overdose and the liver was perfused through the portal vein using a peristaltic pump at a flow rate of 14 ml/min, first with pre‐warmed washing buffer (1 % HEPES- Hanks' balanced salt solution, 1 mM EGTA) and then, with a pre-warmed perfusion buffer (1 % HEPES- HBSS, 2.5 mM CaCl_2_, 0.5 mg/ml collagenase). In order to allow outflow of the solutions, the cava vein was cut. After perfusion, the liver was excised, cut and placed in a pre-warmed digestion solution (1 % HEPES- HBSS, 2.5 mM CaCl_2_, 0.05 mg/ml collagenase, 0.03 mg/ml DNAse) for 5 min in a water bath at 37 ºC. Digested tissue was filtered through a 100 µm cell strainer to eliminate undigested tissue remnants and cell suspension was centrifuged at 70 g for 1 min at 4 ºC. The supernatant contained non-parenchymal cells (NPC), whereas hepatocytes were found in the pellet. HSCs were purified from the NPC fraction after 14 % Nycodenz gradient (Sigma-Aldrich). Following centrifugation at 2500 rpm without brake for 15 min at room temperature, two interphases were obtained. The upper interphase was enriched in HSC and the lower interphase contained polymorphonuclear cells and EC. The lower interphase was seeded in petri dishes and incubated for 35 min in a humid 5 % CO_2_ atmosphere in order to enhance EC purity by selective adherence time of PMN.

***Immunodetection of α-SMA, PTTG1 and DLK1.*** Liver sections from fibrotic rats underwent microwave antigen retrieval to unmask antigens hidden by cross-linkage occurring during tissue fixation. Endogenous peroxidase activity was blocked by hydrogen peroxide pretreatment for 10 min and with 5 % goat serum for 45 min. The sections were then stained with mouse anti-α-SMA (1:1200; Dako Denmark A/S, Glostrup, Denmark), rabbit anti-DLK1 (1/250 Abcam, Milton, Cambridge, UK), or rabbit anti-PTTG1 (1/100 Invitrogen, Waltham, Massachusetts, USA) and the first was incubated for 1 h at room temperature and the two latter were incubated overnight at 4 ºC. The LSAB 2 System-HRP (Dako Denmark A/S) was used for antigen detection and antigen visualization was achieved with streptavidin peroxidase and counterstained with hematoxylin. As negative controls, immunostaining was performed without the first antibody. The area of positive staining was visualized using a digital microscope (Eclipse E600; Nikon, Tokyo, Japan) in 16 random fields per animal. The percentage of immunostained α-SMA/fields areas of digital photomicrographs was then quantified.

***Western Blot Analysis of DLK1, TGFβ, TNFα and AII.*** Hepatic tissue from fibrotic and control rats was individually homogenized as described previously (2). To detect DLK1, TGFβ, TNFα and AII, 40 μg of total denatured proteins were loaded on a 10 % (DLK1) and 12 % (TGFβ, TNFα and AII) SDS-polyacrylamide gel (Mini-PROTEAN III; Bio-Rad Laboratories, Hercules, CA). Gels were transferred for 2 h at 4 ºC to nitrocellulose membranes of 0.45 μm for DLK1, AII and TNFα, and to 0.2 μm for TGFβ, which were stained with Ponceau S Red as a control for protein loading. Thereafter, membranes were blocked with 5 % non-fat milk for TGFβ and AII and with 5 % and 1 % bovine serum albumin for TNFα and DLK1, respectively, in TTBS buffer at room temperature for 2 h. Then, they were incubated overnight at 4 ºC with rabbit monoclonal anti-DLK1 (1:1000 dilution; Abcam, Cambridge, UK), polyclonal anti-TGFβ, (1:1000 dilution; Abcam, Cambridge, UK), polyclonal anti-AII (1/500 Santa Cruz, Dallas, TX) or polyclonal anti-TNFα antibodies (1/1000 dilution; Cell Signaling Technology, Danvers, MA), followed by incubation with a donkey anti-rabbit horseradish peroxidase-conjugated secondary antibody (1:2000; Amersham Biosciences, GE Healthcare, Piscataway, NJ, USA). The bands were visualized by chemiluminescence (Luminata Forte Western HRP substrate; EMD Millipore, Billerica, MA, USA).

| **Antibody** | **Catalog number** | **Brand** |
| --- | --- | --- |
| Anti-DLK | ab21682 | Abcam |
| Anti-AII | sc-20718 | Santa Cruz Biotechnology INC |
| Anti-TNF alpha | 3707 | Cell Signaling Technology |
| Anti-TGF beta | ab66043 | Abcam |

***Messenger expression of PTTG1, DLK1 and COL1α1 in human liver tissue.*** To assess whether *PTTG1* mRNA is over expressed in human fibrotic liver, this parameter as well as *COLIα1* and *DLK1* mRNA were assessed in a group of 12 samples obtained by liver resection from patients with cirrhosis associated with hepatitis C virus infection. In addition, we analyzed liver biopsies from 7 non-cirrhotic patients. Non-cirrhotic samples were obtained from fragments of colon metastatic resections before vascular clamping. Patients provided written informed consent for medical research according to the principles of the Declaration of Helsinki. Total RNA was extracted as previously described and 1 μg of total RNA was reverse transcribed using a complementary DNA synthesis kit (High-Capacity cDNA Reverse Transcription Kit, Applied Biosystems, Foster City, CA). The primers and probes for human *PTTG1 (probe#22* left: 5’-GCCTCTCATGATCCTTGACG-3’, right: 5’-GCTTGAAGGAGACTGCAACA-3’)*, DLK1* (*probe#68* left: 5’-GACGGGGAGCTCTGTGATAG-3’, right: 5’-TCATAGAGGCCATCGTCCA-3’), *COL1α1* (*probe#* left: 5’-AATCCTCGAGCACCCTGA-3’ right: 5’-CCCCTGGAAAGAATGGAGAT-3’), and hypoxanthine-guanine phosphoribosyltransferase (*HPRT*), used as an endogenous standard (*probe#73* left: 5’-TGACCTTGATTTATTTTGCATACC-3’, right: 5’-CGAGCAAGACGTTCAGTCCT-3’) were designed according to human *PTTG1, DLK1*, *COL1α1* and *HPRT* sequences (GenBank NM_001282382.1, NM_003836.6, NM_000088.4. and NM_000194.2, respectively).

***Cell culture and treatments.*** CC-1 cells, an adult rat hepatocyte cell line (3), were a generous gift from Dr. J. Clària. Cells were grown at 37 °C in a 5 % CO_2_ atmosphere in Eagle's minimum essential medium media supplemented with 2 mM of glutamine, 1 % nonessential amino acids, penicillin (50 U/ml), streptomycin (50 μg/ml), 20 mM HEPES and 10 % fetal bovine serum. Cells were plated (1 x 10^5^ per well) in 24-well plates (MilliporeSigma, Massachusetts, USA) and grown until 70 % confluence. The medium was replaced by serum free medium 24 h before transfection.

***In vitro Pttg1 interference.*** CC-1 cells were transfected with a final concentration of 20 nM of Silencer Select PTTG1 Pre-Designed siRNA (*Pttg1* siRNA), Silencer Select GAPDH positive control siRNA (C^+^siRNA) or Silencer Select Negative control No 1 siRNA (C- siRNA). Transfections were performed using Lipofectamine RNAiMAX Reagent according to the manufacturer’s instructions (Life Technologies, Grand Island, NY, USA). Cells were incubated in Opti-MEM Reduced Serum Media (Invitrogen Life Technology) with or without Silencer Select siRNA- Lipofectamine RNAiMAX complexes for 24, 48 and 72 h. All transfections were performed in duplicate. The efficacy of gene silencing was assessed by quantitative real-time PCR analysis. *Pttg1 siRNA* is a 21 bp duplex deoxyribonucleotide with a sense strand corresponding to nucleotides 392 to 410 of the reported rat *Pttg1* mRNA sequence. All siRNAs were designed and synthesized by Ambion (Life Technologies LTD, Leicestershire UK).

***mRNA expression of Dlk1 and Pttg1 in isolated and cultured cell lines.*** Total RNA from HEP, HSC, EC, and CC-1 cells was extracted using TRIzol (TRI Reagent; Sigma-Aldrich, St. Louis, MO, USA). One microgram of total RNA was reverse transcribed as previously described. Specific primers and probes used for the different genes studied were designed to include intron spanning using the Universal Probe Library Assay Design Center through the ProbeFinder v2.45 software (Roche Diagnostics, Indianapolis, IN. <https://www.roche-applied-science.com/sis/rtpcr/upl/index.jsp>). *Pttg1* (probe#68; left: 5’-AGTCTACTAAGACACAAGGCTCTGC-3’, right 5’-CAGGCAGGTCAAAACTCTCA-3’), *Dlk1* (probe#76; left: 5’-CCTGTGTGAGAAGTGCGTA-3)’, *Hprt* (probe#95; left: 5’-GACCGGTTCTGTCATGTCG-3’ right: 5’-ACCTGGTTCATCATCACTAATCAC-3’) was used as the reference gene. The primers were designed according to rat sequences (GenBank NM_022391.3, NM_053744.1, NM_012583.2 respectively). Real time quantitative PCR was analyzed in duplicate and performed with the Light Cycler 480 (Roche Diagnostics). Ten µl volume reactions of diluted 1:8 cDNA, 200 nM primer dilution, 100 nM pre-validated 9-mer probe (Universal ProbeLibrary, Roche Diagnostics) and FastStart TaqMan Probe Master (Roche Diagnostics) were used in each PCR reaction. The fluorescence signal was captured during each of the 45 cycles (denaturizing 10 s at 95 ºC, annealing 20 s at 60 ºC and extension 1 s at 72 ºC). *Hprt* was used as a reference gene for normalization and water was used as a negative control. Relative quantification was calculated using the comparative threshold cycle, which is inversely related to the abundance of mRNA transcripts in the initial sample. The mean CT of duplicate measurements was used to calculate ΔCT as the difference in CT for target and reference. The relative quantity of product was expressed as the fold-induction of the target gene compared with the reference gene according to the formula 2^-ΔΔCT^, where ΔΔCT represents ΔCT values normalized with the mean ΔCT of control samples

***Droplet digital PCR (ddPCR) analysis***. The total number of transcripts of two candidate genes *Pttg1* (assay ID: dRnoCPE5168832, Bio-Rad, Hercules, California, USA) and *Dlk1* (assay ID: dRnoCPE5166190) and a reference gene *Hprt1* (assay ID: dRnoCPE5167187) of isolated primary cirrhotic cells were quantified using the ddPCR platform (QX200; Bio‐Rad). In addition, *Pttg1* and *Hprt1* absolute transcripts were also evaluated in organs (liver, spleen, lung, kiney, heart, aorta, and brain) from control and cirrhotic rats. The total 20 µl reaction mixture included 10 µl of 2x ddPCR Supermix for probes (no dUTP), 1 µl of 20x target primer/probes (FAM), 1 µl of 20x reference primer/probes (HEX), 1 µl of cDNA from 25 ng RNA, and 7 µl of RNase/DNase free water. Each ddPCR reaction mixture and 70 µl of droplet generation oil were carefully loaded to a DG8 cartridge, which was covered with Droplet Generator Gasket (Bio-Rad) and transferred into the QX200 Droplet Generator (Bio‐Rad) to generate a maximum of 20,000 droplets from each sample. Then, 40 µl of droplets were transferred to a 96‐well PCR plate for amplification using the C1000 Touch PCR thermal cycler (Bio‐Rad). Each reaction was performed in duplicate. The PCR conditions were as follows: enzyme activation (95˚C, 10 min), 40 cycles of denaturation (94 ˚C, 30 sec) and annealing (55˚C, 1 min), enzyme deactivation (98˚C, 10 min), and final hold (4˚C). The temperature ramping rate was 2˚C/sec. Following PCR, the plates were directly analyzed with the QX200 Droplet Reader (Bio-Rad). The data were processed using Quanta Soft Analysis Pro 1.0.596 (Bio-Rad). Samples with >10,000 droplets were considered for further analysis. The individual threshold for each sample was automatically calculated by the software. QuantaSoft software provides concentration results in copies of target per µl.

***Hepatic messenger expression of fibrosis related genes***. Liver samples from treated and untreated animals were also fixed in 10 % buffered formalin for further hematoxylin and eosin and immunostaining analysis. Total RNA was extracted using a commercially available kit (RNAeasy, Qiagen, Germany) and reverse transcribed. A group of selected fibrosis-related genes was analyzed, including: *Pttg1*, *Dlk1*, *Pdgfrβ* (probe#69; left: 5’-GCGGAAGCGCATCTATATCT-3’, right 5’-GCGGAAGCGCATCTATATCT-3’), *Tgfβr1*(probe#53; left 5’-AAGGCCAAATATTCCCAACA- 3’, right 5’- ATTTTGGCCATCACTCTCAAG-3’), *Col1α1* (probe#95; left 5’-AGACCTGGCGAGAGAGGAGT-3’, right 5’-ATCCAGACCGTTGTGTCCTC-3’), Collagen III α1 (*Col3α1*) (probe#49; left: 5’-TCCCCTGGAATCTGTGAATC-3’, right 5’-TGAGTCGAATTGGGGAGAAT-3’), tissue inhibitor of matrix metalloproteinases type 1 (*Timp1*) (probe#95; left 5’-CATGGAGAGCCTCTGTGGAT-3’, right 5’-TGTGCAAATTTCCGTTCCTT-3’), tissue inhibitor of matrix metalloproteinases type 2 (*Timp2*) (probe#73; left 5’-GACAAGGACATCGAATTTATCTACAC-3’, right 5’-CCATCTCCTTCCGCCTTC-3’), *Mmp2* (probe#60; left 5’-CTCCACTACGCTTTTCTCGAAT-3’, right 5’-TGGGTATCCATCTCCATGCT-3’) and matrix *Mmp9* (probe#53; left: 5’-CCTGAAAACCTCCAACCTCA-3’, right: 5’-GAGTGTAACCATAGCGGTACAGG-3’). *Hprt* ) was used as the reference gene. Primers were designed according to rat sequences (GenBank NM_022391.3, NM_053744.1, NM_031525.1, NM_012775.2, NM_053356.1, NM_032085.1, NM_053819.1, NM_021989.2, NM_031054.2, NM_031055.1 and NM_012583.2 respectively). Real Time quantitative PCR was performed as described above.

***DLK1 quantification and other measurements.*** Serum DLK1 levels were determined by an enzyme-linked immunosorbent assay (ELISA Kit for Rat DLK1, USCN Life Science Inc., Wuhan, China) that measures the serum levels of the 50 kDa larger soluble form of the DLK1 protein. The assay was conducted according to the manufacturer’s instructions and determined at a wavelength of 450 nm (FLUOstar OPTIMA; BMG LABTECH, Ortenberg, Germany). Standard parameters of liver and renal function were measured in the BS-200E Chemistry Analyzer (Mindray Medical International Ltd, Shenzhen, China).

**
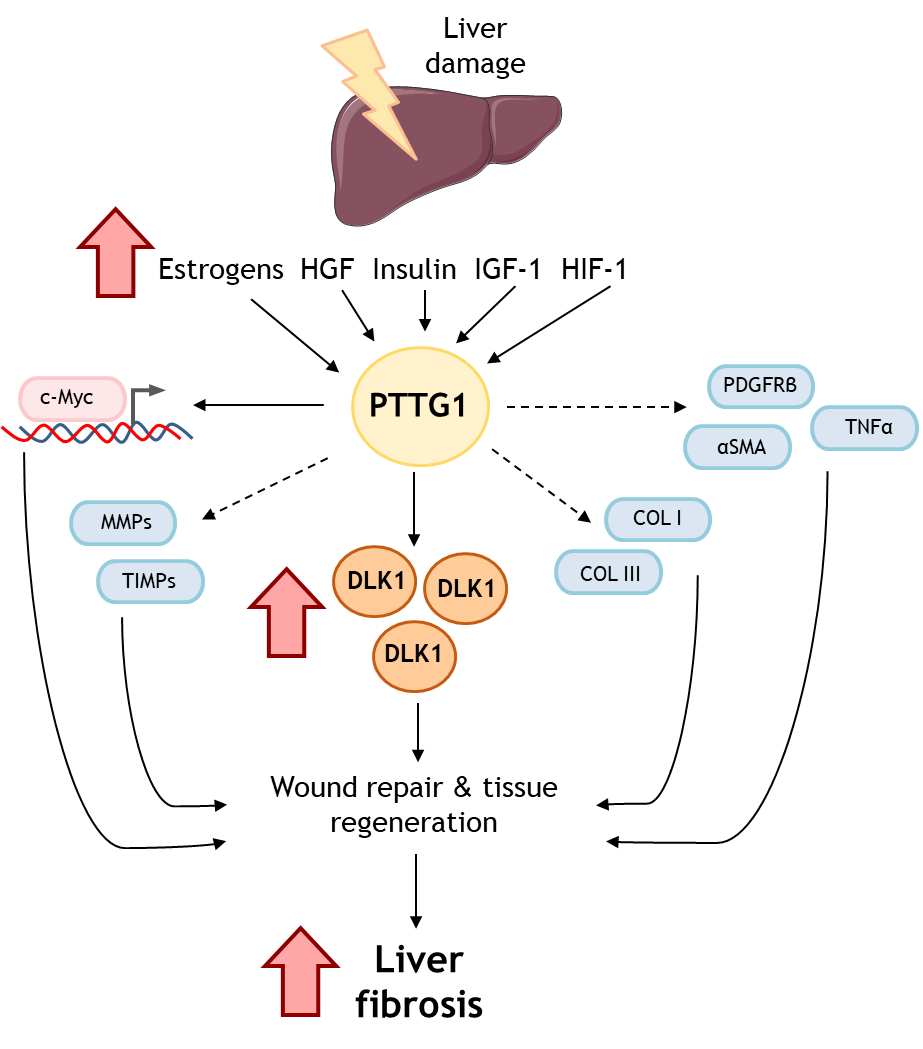
 *Supplementary graphical model.***

**Proposed mechanism underlying PTTG1-induced promotion of liver fibrosis.** Liver injury increases the synthesis of mediators such as estrogens, hepatocyte growth factor (HGF), insulin, insulin growth factor 1 (IGF-1), and hypoxia inducible factor 1 (HIF-1), which, in turn, induce the expression of *Pttg1*. *Pttg1* acts as a transcriptional activator of c-myc. Furthermore, *Pttg1* has an indirect effect on increasing the expression of metalloproteinases (MMPs), tissue inhibitors of metalloproteinases (TIMPs), collagens I (COL I) and III (COL III), platelet derived growth factor receptor beta (PDGFRβ), tumor necrosis factor alpha (TNFα), and alpha-smooth muscle actin (αSMA). Importantly, *Pttg1* increases *Dlk1* expression, which is involved in wound repair and tissue regeneration. Activation of these mechanisms finally results in accentuation of liver fibrosis. Continuous arrows indicate a direct effect, whereas discontinuous arrows denote an indirect effect.

**Supplementary Table 1:** **Body weight, standard liver function tests and serum electrolyte values in the rats included in the study**.

|  | **Control**  **(n=13)** | **Mild**  **Fibrosis (n=6)** | **Severe**  **Fibrosis (n=8)** | **Cirrhosis**  **(n=11)** |
| --- | --- | --- | --- | --- |
| **Fibrotic area (%)** |  | < 6% | 6 - 11% | > 11% |
| **Body weight (g)** | 489 + 8 | 432 + 14 ^*^ | 373 + 56 ^*^ | 396 + 13 |
| **Alanine Transaminase (U/l)** | 44 + 3 | 280 + 107 ^**^ | 618 + 240 ^***^ | 749 + 415 ^***^ |
| **Aspartate Transaminase (U/L)** | 87 + 5 | 195 + 50 ^##^ | 571 + 204 ^#^ | 1454 + 366 ^***^ |
| **Total bilirubin (mg/dl)** | 0.00 + 0.00 | 0.05 + 0.02 ^##^ | 0.34 + 0.20 ^*^ | 2.03 + 0.33 ^***^ |
| **Total proteins (g/l)** | 63.9 + 1.7 | 59.2 + 5.8 | 56.1 + 2.2 ^#^ | 42.4 + 2.1 ^***^ |
| **Albumin (g/l)** | 37.1 + 0.6 | 31.8 + 1.1 | 33.2 + 1.3 ^#^ | 23.2 + 1.2 ^***^ |
| **Gamma-glutamyltransferase (U/l)** | 0.11 + 0.06 | 0.53 + 0.18 ^#^ | 4.51 + 1.90 ^**^ | 12.71 + 2.02 ^***^ |
| **Creatinine (mg/dl)** | 0.56 + 0.01 | 0.50 + 0.06 ^#^ | 0.48 + 0.03 ^#^ | 0.69 + 0.06 |
| **Na^+^ (mEq/l)** | 143.0 + 1.1 | 142.4 + 1.2 | 142.9 + 0.9 | 146.0 + 2.1 |
| **K^+^ (mEq/l)** | 5.41 + 0.12 | 4.99 + 0.27 ^#,+^ | 4.27 + 0.17 ^***^ | 4.19 + 0.15 ^***^ |

*p<0.05, ^*^p<0.01, ***p<0.001 compared with control group; ^#^p<0.05, ^##^p<0.01 compared with cirrhotic group; p< 0.05 compared with severe fibrosis. One-way ANOVA with the Newman-Keuls post hoc test and Kruskal-Wallis test with the Dunn post hoc test when appropriate. Results are given as means ± S.E.

**Supplementary references**

1. Jiménez W, Parés A, Caballería J, Heredia D, Bruguera M, Torres M, *et al*. Measurement of fibrosis in needle liver biopsies: evaluation of a colorimetric method. Hepatology.1985; 5(5):815-818.
2. Muñoz-Luque J, Ros J, Fernández-Varo G, Tugues S, Morales-Ruiz M, Álvarez CE, *et al*. Regression of fibrosis after chronic stimulation of cannabinoid CB2 receptor in cirrhotic rats. J Pharmacol Exp Ther.2008; 324(2):475-483.
3. López-Parra M, Titos E, Horrillo R, Ferré N, González-Périz A, Martínez-Clemente M, *et al*. Regulatory effects of arachidonate 5-lipoxygenase on hepatic microsomal TG transfer protein activity and VLDL-triglyceride and apoB secretion in obese mice. J of Lipid Res.2008; 49(12):2513-2523.
